# Successful management of life-threatening post-COVID-19 cryptosporidiosis in a renal transplant patient: a case report

**DOI:** 10.11604/pamj.2023.45.10.39548

**Published:** 2023-05-04

**Authors:** Oadi Nawaf Shrateh, Afnan Jobran, Momen Ahmad Zaid, Muttaz Saleh

**Affiliations:** 1 Faculty of Medicine, Al-Quds University, Jerusalem, Palestine,; 2 Department of Internal Medicine, Palestinian Medical Complex, Ramallah, Palestine

**Keywords:** Cryptosporidium, renal transplant, zoonotic species, COVID-19, case report

## Abstract

Worldwide, Cryptosporidium spp. is a common parasite that affects domestic and wild animals, including humans, and causes diarrhea in both immunocompetent and immunocompromised hosts. The fecal-oral pathway accounts for the majority of its transfer. Although C. parvum and C. hominis are the most common zoonotic species in humans, other zoonotic species can also infect immunocompetent and immunocompromised people. Patients undergoing renal transplants are more likely to contract cryptosporidiosis, which can cause severe and potentially fatal diarrhea. A 41-year-old male patient who presented to the emergency department complained of a sudden onset, severe and continuous fatigue, and a feverish sensation of two-day duration. Two days prior to the current admission, the patient started to complain of weakness affecting his whole body, as well as a fever of 39°C and continuous yellowish diarrhea occurring 4-5 times daily without blood. Stool analysis revealed a cryptosporidium infection. The patient underwent surgery for kidney transplantation. The donated kidney was the left one from his brother and was attached to the patient´s right groin. As illustrated by our example, cryptosporidiosis should be considered a significant cause of acute, persistent, watery diarrhea in immunocompromised kidney transplant recipients. Patients undergoing renal transplants should be instructed to wash their hands frequently, stay away from young animals, sick people, and swimming pools in order to lower their risk of infection.

## Introduction

Diarrhea is brought on by the protozoan *Cryptosporidium spp*. after drinking tainted water or food. The host's immunological state affects the duration and severity of the disease [1]. Infections can cause severe and/or widespread disease in immunocompromised individuals, whereas they are self-limited in immunocompetent hosts. Acquired immunodeficiency syndrome (AIDS) patients have been found to have cryptosporidiosis, which has more recently been identified as a cause of severe and chronic diarrhea in solid organ transplant (SOT) patients [2]. Within three years of the transplant, one-fifth of the SOT population will experience diarrhea. By far, clostridium difficile and norovirus are the pathogens most frequently implicated in cases of chronic diarrhea. Cryptosporidiosis, however, may be the cause of diarrhea in this situation [3]. Most reports only cover a single patient or a limited number of patients. One study in an endemic region of India found that 28% of transplant patients' diarrhea was caused by *Cryptosporidium spp*. (34 patients). The clinical symptoms, prognosis, and course of treatment for cryptosporidiosis in SOT patients are still not well understood [3].

## Patient and observation

**Patient information and clinical findings:** our patient is a 41-year-old male patient who presented to the emergency department complaining of feverish sensation and fatigue for two-day duration. Past medical history is positive for controlled hypertension for 15 years and chronic kidney disease for 3 years with anemia and a creatinine level of 13 mg/dl. Past surgical history is significant for open heart surgery in tricuspid valvuloplasty.

**Timeline of current episode:** on the 10^th^ of August 2022, the patient underwent surgery for kidney transplantation. The donated kidney was the left one from his brother and was attached to the patient´s right groin. He was started on 1 mg of tacrolimus three times daily, mycophenolate mofetil 500 mg once daily, and prednisolone 500 mg twice daily. Shortly after, a complication occurred where the ureter became blocked, and the surgeon had to put a stent in it to allow urine to flow into the bladder. The stent was removed 21 days after it was placed. Then the ureter was blocked once again, and a stent was placed for 2 months. On November 11^th^ 2022, the patient was admitted to the hospital when his creatinine reached 4. The stent was removed, and a biopsy of the kidney was performed with normal results. After 1 week of hospitalization, the patient was infected with the coronavirus disease (COVID-19), which was managed appropriately followed by discharge from the hospital. Nowadays, the patient experiences a sudden onset, severe and continuous fatigue, and a feverish sensation of two-day duration. Two days prior to the current admission, the patient started to complain of weakness affecting his whole body, as well as a fever of 39°C and continuous yellowish diarrhea occurring 4-5 times daily without blood.

**Diagnostic assessment and diagnosis:** stool analysis revealed a cryptosporidium infection.

**Therapeutic interventions:** the patient was prescribed nitazoxanide 500 mg, twice daily for 3 days. The patient also complains of daily night sweats and loss of appetite, occasional shortness of breath, and non-projectile vomiting of yellowish, undigested gastric contents with a volume of 1 cup, accompanied by nausea.

**Follow-up and outcome of interventions:** the patient achieved successful recovery of cryptosporidium infection with the resolution of diarrhea.

**Patient perspective:** the patient was satisfied with the care he received during his hospitalization course and during follow-up visits.

**Patient consent:** written informed consent was obtained from the patient for the publication of this case report. A copy of the informed consent is available for review by the editor-in-chief of this journal on request.

## Discussion

While stool microscopy has traditionally been the preferred diagnostic technique for assessing suspected parasitic gastrointestinal illnesses, nucleic acid detection techniques are being used more frequently due to claims that they have higher sensitivity, specificity, and quick turnaround times. Given the large range of nucleic acid assays available, their various methodologies, the small number of positive clinical specimens for focused comparisons, and other factors, the association between different approaches is still unclear [4]. The latter is especially true in areas with low frequency, which are frequently industrialized nations where such assays are more generally accessible. It's true that nucleic acid-based assays frequently detect pathogen deoxyribonucleic acid (DNA) in low quantities or even that of nonviable bacteria, raising questions about therapeutic value in such circumstances [2]. There aren't many studies, if any, that directly correlate clinical outcomes of different diagnostic techniques. Although it does not distinguish between species, the gastrointestinal panel (GIP) has two assays for the identification of around 23 different *Cryptosporidium spp*. including *Cryptosporidium hominis* and *Cryptosporidium parvum*. The extremely rare species of *Cryptosporidium xiaoi, Cryptosporidium ryanae*, and bovis are not picked up by it [5]. Despite being examined with only 18 positive clinical specimens, GIP's sensitivity and specificity for identifying cryptosporidiosis were 100% and 99.6%, respectively, in one of the biggest multicenter evaluations. Real-time polymerase chain reaction was used as the comparative assay in this investigation (PCR) [6].

The immunological health of the host affects the intensity and length of human cryptosporidium infections. Immunocompetent patients have self-limiting disease, whereas immunosuppressed patients, particularly those with T-cell loss, frequently have chronic and severe cryptosporidiosis with a risk of developing extra-intestinal disease. Post-transplant cryptosporidiosis with diarrhea is a common complication in recipients of renal transplants. From 2006 to 2009, there were 69.3% of clinically evident cryptosporidiosis cases recorded in France that involved immunocompromised individuals, and 16.5% of those cases involved recipients of solid organ or stem cell transplants [7]. Infections were found to be the main cause of diarrhea in one report from a juvenile renal transplant center, with *Cryptosporidium spp*. being identified in 11% of 199 cases [8]. According to reports, renal transplant patients in Poland and India had *a Cryptosporidium spp*. frequency of 18.8 and 20%, respectively [9]. In contrast to immunocompetent patients, recipients of solid-organ transplants were more frequently affected by *Cryptosporidium spp*. infections that resulted in profuse watery diarrhea [9].

Estimates of the incidence of SARS-CoV-2 infection in kidney transplant patients depend on the availability of SARS-CoV-2 PCR testing, just as epidemiological research in any other population. Such testing was frequently restricted to hospitalized patients in the early stages of the pandemic. However, a number of investigations discovered that kidney transplant recipients had a greater frequency of SARS-CoV-2 infection than the overall population [10]. The causes of this higher incidence are unknown, but they may be related to the high numbers of unavoidable health and social care interactions, susceptibility to infection as a result of immunosuppression, greater access to testing because of the higher risk of serious illness, or because of relationships with healthcare professionals [10]. Additionally, analyses point to the possibility that transplant recipients may experience prolonged viral shedding after infection, which could result in PCR tests having better sensitivity than it would in the general population.

## Conclusion

In immunocompromised kidney transplant recipients, cryptosporidiosis should be taken into account as a substantial cause of acute, persistent, watery diarrhea, as demonstrated by our case. Renal transplant patients should be advised in hospitals or daycare facilities to reduce their risk of infection by washing their hands frequently and avoiding contact with young animals, sick people, and swimming pools. Additionally, patients with protracted diarrhea in the renal transplant unit should be isolated and screened for cryptosporidium. Patients with kidney transplants are at a greater risk of diarrhea caused by cryptosporidium; hence, species identification using molecular biology is crucial. First, to get rid of potential nosocomial infection in patients with whom the nephrology department's consultations will frequently come into contact; second, to find the source of contamination and get rid of it to stop any potential recontamination.
